# Embracing generative AI in health care

**DOI:** 10.1016/j.lanepe.2023.100677

**Published:** 2023-07-03

**Authors:** The Lancet Regional Health – Europe

Generative artificial intelligence (GenAI), including Chat Generative Pre-training Transformer (ChatGPT; developed by OpenAI, and Bard (developed by Google), marks a technological renaissance with exponential future potential that has surpassed previous technological revolutions brought about by inventions such as the internet, search engines, and smartphones. In contrast to contemporary artificial intelligence (AI) tools, GenAI has the ability to create or generate new original content such as text, images, or even music, thus not just simply mimicking human intelligence, but surpassing it.

To date, AI technologies have had limited, yet considerable, applications in health care. Such technologies have been used to improve the analysis of medical images such as x-rays, CT scans, and MRIs for disease diagnosis; for extracting and analysing information from electronic health records; for personalised medicine; for remote monitoring with wearable devices, sensors, and home monitoring systems; and for drug discovery and development.

In contrast, GenAI has the potential to transform clinical workflows and the way doctors work. For example, at the basic level, GenAI can help health-care professionals interpret data such as a patient's medical history, imaging records, genomics, or laboratory results with a simple query, even if the information is stored across different formats and locations. For instance if a physician requires information about a cohort of female patients aged 45–55 years, and needs access to mammograms and medical charts, they can enter this query into the search tool instead of seeking out each element separately.

However, the leap in technological progress has been driven by the ability of GenAI to generate synthetic data, such as medical images that can be used to augment training data and create diverse datasets for research and medical training. Similarly, synthetic medical data can address the issues of data scarcity and privacy concerns. Furthermore, GenAI models can also accelerate drug development by generating novel molecular structures with desired properties. It is entirely conceivable that GenAI could eventually replace the need for most clinical trials and laboratory experiments, thus accelerating clinical and scientific developments at an unprecedented pace**.**

The potential of GenAI in revolutionising health care is limitless. Recognising this potential, on June 7, Google Cloud announced a collaboration with Mayo Clinic, which excels at leveraging AI in health care using electronic health records. This represents an important collaboration between two market leaders to utilise GenAI within health care. More investment is expected through the partnership of technology companies with health-care organisations, which would eventually lead to changes and improvement in the way health care is delivered.

Considering the rapid pace of technological developments, the most important concern regarding GenAI in health care is ensuring precision of results, diagnosis, and analysis, since inaccuracy can be detrimental for patients. Since GenAI technologies rely on the data they are trained on, and much of the existing data are already heavily biased due to poor diversity, especially in clinical and genetic domains, the likelihood of misinformed biased results could be high in the clinical setting. For use in personalised medicine, training data for GenAI must be free of gender, racial, social, or religious biases. Other immediate concerns include ensuring patient confidentiality when using GenAI, validation and reliability of output generated, accountability, and liability.

Although there are pertinent concerns regarding the use of GenAI in health care, resisting its transformative potential would be futile and non-pragmatic. We need to embrace this paradigm technological shift, but with caution and strict regulation. National and global regulatory bodies are struggling to adapt to fast-paced developments around GenAI, as no one really knows what to expect in the future. It is a challenging task for any regulatory authority to envision the evolution of GenAI to cover all aspects of regulation.

The European Union (EU) has taken the first step towards AI regulation. On June 14, EU lawmakers passed landmark AI regulation under EU's AI Act—one of the world's first formal rules for the technology—requiring GenAI systems to be reviewed before commercial release. The regulation is far from becoming a law and has received criticism for not taking into account the opinions of human rights campaigners and the individuals who developed the technology, not only in the commercial sector, but also in universities and in the open-source communities.

It is too early to predict what this regulation would mean for the future—will Europe lag behind other regions or set the precedent for future rules and standards globally for GenAI? What is clear is that the world is changing, and we need to change the way we think about health care by embracing the potential of GenAI to improve efficiency of health-care delivery and decrease the burden on health-care workers.

Health-care regulators in particular need to adapt to advancements in GenAI to ensure patient safety, privacy, and ethical application. Additionally, there is a need for a specific regulatory body to oversee the use of GenAI in health care and research per se, similar to the safety and ethical regulatory bodies that regulate clinical trials, drug approvals, and preclinical studies.

